# An Emerging Approach for Optimization of Cow Ghee as an Ointment Base in Combination With Selected Conventional Bases

**DOI:** 10.7759/cureus.36556

**Published:** 2023-03-22

**Authors:** Monali B Wawre, Deepak Khobragade, Dharmendra Mundhada

**Affiliations:** 1 Pharmacy, Datta Meghe College of Pharmacy, Wardha, IND; 2 Pharmacy, Agnihotri College of Pharmacy, Wardha, IND

**Keywords:** ayurvedic, free fatty acid, topical, conventional bases, cow ghee

## Abstract

Background

*Cow ghee* is a pure and clean animal fat derived from milk and is often recognized as clarified butter. It is used in Ayurvedic medicine as an excellent base for preparing various formulations due to its ability to penetrate deep tissue and be easily absorbed. Cow ghee possesses antioxidant, antibacterial, anti-inflammatory, and antiseptic properties, making it beneficial for treating skin-associated problems. When applied externally, ointment bases are semisolid preparations for use on the skin or mucous membranes. They are classified into four categories: Hydrocarbon, Absorption, Water-removable, and Water-soluble. In this study, ointment bases were formulated and evaluated using cow ghee and selected conventional ones.

Materials and Methods

Ointment bases like Cetostearyl alcohol, stearic acid, glyceryl monostearate, soft white paraffin, soft yellow paraffin, paraffin wax, white beeswax, and wool fat were obtained from SD fine chem manufacturer Ltd., Mumbai. Cow ghee was obtained from the Go Vigyan, Anusandhan Kendra, Nagpur. The ointment bases were prepared using pharmacopeia procedures. Cow ghee was used as a base in the preparation of ointment bases with different concentrations than conventional bases. Stability testing was performed per International Conference on Harmonization(ICH) guidelines and various physicochemical parameters like color, appearance, odor, consistency, pH, Spreadability Extrudability, loss on drying, solubility, and washability.

Results

The ointment bases formulated using cow ghee in combination with selected conventional ointment bases were found to be stable. They exhibited desirable characteristics like non-greasy, attractive appearance, and suitability for various medications and supporting substances. The cow ghee-based ointment bases also showed good spreadability, extrudability, and solubility, indicating their effectiveness as carriers for active components.

Conclusion

The study demonstrates the potential of cow ghee as a natural ointment base for the preparation of various Ayurvedic formulations. The ointment bases formulated using cow ghee in combination with conventional ointment bases were stable and exhibited desirable physicochemical properties. Thus, using cow ghee as an ointment base can provide a cost-effective and easily accessible alternative for therapeutic use or as a carrier of active components.

## Introduction

Cow ghee

The pure and clean animal fat derived from milk is treated as ghee and is often recognized as clarified butter. Cow ghee is used in long-standing Indian development itself. Cow ghee does not spoil longer because it does not include water. Cow ghee's purest form is thin, greasy, and devoid of water, lactose, and other milk particles. For the grounding of Ayurvedic medicines, cow ghee is viewed as an admirable base. Cow ghee is a perfect base for preparing ayurvedic dosage form because of its capacity to penetrate the deeper tissue, cross the cell membrane, and be easily absorbed. According to Ayurveda, ghee's efficiency and medicinal qualities improve as it ages and has a bitter flavor. Five to ten years older ghee is highly favored in several Ayurvedic formulas. Also, it describes cow ghee as an effective medium for delivering medications into the deeper layers of the body's tissue. Lipids are found in human cell membranes, and cow ghee has a highly lipophilic effect. This makes it easier for cow ghee to deliver the medicines to the desired cellular level [1].

In Ayurveda, cow ghee is a natural ointment used in the preparations of medicines as an ointment base [2,3]. Cow ghee has antioxidant properties as it contains fat-soluble Vit E and beta carotene [1]. It also possesses antibacterial, anti-inflammatory, and antiseptic properties that are beneficial in treating various skin-associated problems. So, it helps treat blisters, inflammatory swellings, and wounds to promote the healing process speedily [4]. A combination of well-chosen conventional ingredients bases and cow ghee is accommodating for burn treatment and other injuries on the skin because of the outstanding healing properties of cow ghee [3]. While selecting cow ghee as a base, another approach is that it is extensively utilized, conveniently accessible, and affordable throughout the region [4].

Ointment bases

When applied externally, the ointment is described as a semisolid preparation intended for use on the skin or mucous membranes. Ointment (semisolid) bases fall into four categories: Hydrocarbon, Absorption, Water removable, and water-soluble. No matter their specific functions, all ointment bases should possess particular qualities. These consist of A. Under distinctive use and storage circumstances, it is chemically and physically stable; B. Nonreactive and suitable for a variety of medications and supporting substances; C. Free from offensive odors; D. No irritating, nontoxic, or no sensitizing; E. Attractive, simple to use, and non-greasy; F. Until it is removed, it remains in contact with the skin ointment bases intended for therapeutic use or as a carrier of active components [5,6].

## Materials and methods

Aim

The persistence of this work is to formulate and evaluate ointment bases using cow ghee in combination with selected conventional ointment bases.

Materials

The ointment bases like Cetostearyl alcohol, stearic acid, glyceryl monostearate, soft white paraffin, soft yellow paraffin, paraffin wax, white beeswax, and wool fat were obtained from SD fine chem manufacturer Ltd, Mumbai. Cow ghee was obtained from the Go Vigyan, Anusandhan Kendra, Nagpur. Based on certain factors, a routine or Pharmacopoeia procedure was used to recognize and authenticate cow ghee. The parameters include solubility, pH, loss on drying, ash content, refractive index, specific gravity, saponification value, acid value, iodine value, moisture content, peroxide value, microbial load, zinc content, vitamin C content, heavy metal analysis3, All of the analytical-grade solvents used for titrations to estimate levels of free fatty acids and peroxide were purchased from Sigma Aldrich Pvt. Ltd. in Mumbai.

Preparation of ointment base

In this procedure, the base components were put in a melting pan at 70°C. After melting, the components were gently swirled while being kept at a temperature of 70°C for about 5 minutes, and then they were continuously cooled to a temperature of 40°C. After being mixed to a smooth consistency, ointments were kept at room temperature (25°C)6. Ointment bases were prepared using pharmacopeia procedures. Later a portion of the lipid is utilized in the procedure and is replaced with cow ghee and prepared ointment bases varying in the concentration of cow ghee.

Cow ghee is used as a base in the ointment bases preparation having different concentrations with conventional bases like Cetostearyl alcohol, stearic acid, glyceryl monostearate, Paraffin wax, soft white paraffin and soft yellow paraffin, white beeswax, and wool fat. Cow ghee and conventional bases are heated separately, bestowing to the declining order of melting point, and mixed.

Stability of ointment base

The organoleptic characteristics were examined, and stability testing was performed per ICH guidelines. The stability of the formulation of ointment bases was evaluated regarding variations in physical and chemical factors expected to impact it. Various physicochemical parameters were carried out, like color, appearance, odor, consistency, pH, spreadability, extrudability, loss on drying, solubility, and washability 7.

Color and appearance

The ointment base was precisely weighed at 2 gm, put into the watch glasses, and evaluated visually by the naked eye by placing it against the white background.

Odour

Accurately weighed 2 gm of ointment base, was taken into watch glasses, and smelled.

Consistency

Smooth, and no signs of greediness were found.

pH

A digital pH meter was used to measure the pH of the bases for ointments. For this, 100 ml of distilled water was used to dissolve 0.5 g of ointment base, and the pH of the mixture was measured.

Spreadability

The spreadability was assessed by sandwiching extra samples between two slides that had been crushed to a uniform thickness by applying a specific weight for a specific time. Spreadability was determined by measuring the time needed to separate the two slides, and better spreadability is the outcome of separating two slides in less time.

The following formula calculated the spreadability.

S=M×L/T

Where S= Spreadability

M= Weight tide to the upper slide

L= Length of glass slide

T= Time taken to separate the slides

Extrudability

The collapsible tube container was filled with the ointment base. The weight of the ointment base needed to extrude 0.5 cm of ointment base ribbon in 10 seconds was used to calculate the extrudability.

Solubility

Soluble in boiling water, ether, chloroform, miscible with alcohol.

Washability

After applying an ointment base to the skin, the ease of washing with water was evaluated.

Non-irritancy Test

A prepared ointment base was applied to a human's skin, and the outcome was monitored.

Homogeneity

Both the ointment base's visual appearance and tactile uniformity were evaluated. The ointment base's appearance was esteemed according to its color, pearlescence, and roughness.

After Feel

A predetermined amount of ointment base was pragmatic, and the amount of residue left after the application was evaluated for emollients and slipperiness.

Stability Study

The physical stability of prepared ointment bases was tested for 45 days at various temperatures, including 2°C, 25°C, and 37°C. Within four weeks, it was discovered that the ointment bases were physically stable at three different temperatures, namely 2°C, 25°C, and 37°C.

Degradation of Product

Under expedited sceneries, the ointment bases were checked for any degradation that might have happened throughout the research period.

Chemical Stability

Due to the inclusion of ghee, ointment bases were assessed for the amount of free fatty acids they emitted, and lipid peroxide values were determined per Indian norms.

## Results

**Figure 1:**
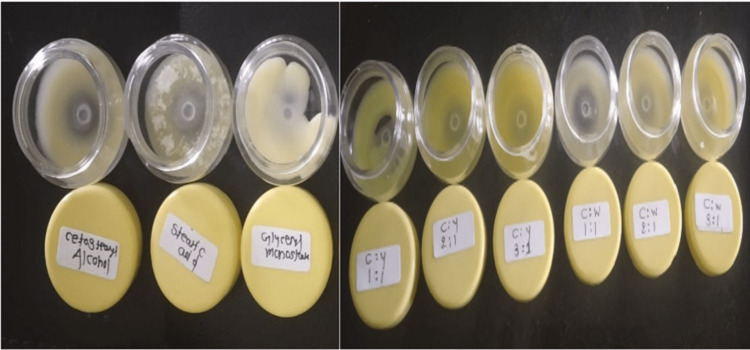
Formulation of Ointment bases with different conventional bases having different concentrations of Cow ghee.

As we know, cow ghee has various properties like antioxidant, anti-inflammatory, wound healing, etc., so in this study, it is used as an ointment base and other ointment bases and evaluated for its compatibility with other ointment bases. We conducted research combining various ghee concentrations with other bases (Table 1, Figure 1) that give Cow ghee's physical and chemical parameters. A total of nine ointment bases were prepared F1 to F9, the composition is given in (Table 2-5), and all these are evaluated in terms of physical and chemical stability.

**Table 1:** Physical and chemical parameters of Cow ghee

Parameters	Values of Cow ghee
Organoleptic properties	
Color	Yellow
Odour	Characteristics, pleasant
Taste	Characteristics
Texture	Oily, Granular
Physical Parameters	
pH	5.9
Melting range	36.5°C - 37.5°C
Moisture content	0.13
Particle size	55.67
Chemical Parameters	
Acid value	0.72
Saponification value	226
Iodine value	38.12
Unsaponifiable matter	0.55
Peroxide value	7.5
Ester Value	224.7

**Table 2:** Composition of ointment bases

Composition	Proportions of Ointment bases and Formulations batches								
	F1	F2	F3	F4	F5	F6	F7	F8	F9
Cow ghee	5 g	5 g	5 g	5 g	5 g	10 g	15 g	10 g	15 g
Cetostearyl alcohol	5 g	-	-	-	-	-	-	-	-
Stearic acid	-	5 g	-	-	-	-	-	-	-
Glyceryl monostearate	-	-	5 g	-	-	-	-	-	-
White soft paraffin	-	-	-	5 g	-	-	-	5 g	5 g
Yellow soft paraffin	-	-	-	-	5 g	5 g	5 g	-	-

**Table 3:** Physicochemical assessment of formulated ointment bases

Physicochemical Parameters	Ointment bases Formulations								
	F1	F2	F3	F4	F5	F6	F7	F8	F9
Color	Off White	Off White	Off White	Pale Yellow	Pale Yellow	Pale Yellow	Off White	Off White	Pale Yellow
Odour	Characteristics	Characteristics	Characteristics	Characteristics	Characteristics	Characteristics	Characteristics	Characteristics	Characteristics
Consistency	Hard	Hard	Hard	Hard	Smooth	Smooth	Hard	Smooth	Smooth
pH	7.345	7.432	7.103	7.223	7.103	7.986	7.345	7.678	7.987
Spreadability (g.cm/Seconds)	9.23	11.09	10.87	11	13.65	17.09	11.98	17.54	16
Washability	Good	Good	Good	Good	Good	Good	Good	Good	Good
Non-irritancy	Non-irritant	Non-irritant	Non-irritant	Non-irritant	Non-irritant	Non-irritant	Non-irritant	Non-irritant	Non-irritant
Stability study (2°C, 25°C, and 37°C)	Less stable	Less stable	Less stable	Less stable	stable	stable	stable	stable	stable

**Table 4:** Values of spreadability of prepared ointment bases F-5, F-6, F-8 and F-9 on different days at various temperatures

Day	Temperature	Spreadability (gm/sec)			
		F5	F6	F8	F9
0^th^	34°C	13.65	17.09	17.54	16
	40°C	13.87	17.65	17.64	16.8
5^th^	34°C	13.63	16.14	18.04	16.04
	40°C	13.84	16.67	18.13	16.5
15^th^	34°C	14.04	17.43	17.09	16.28
	40°C	14.10	17.79	17.55	16.43
30^th^	34°C	13.94	17.34	17.45	16.03
	40°C	14.06	17.92	17.59	16.34
45^th^	34°C	14.23	17.02	18.07	16.64
	40°C	14.29	17.24	18.21	16.83

**Table 5:** Free fatty acid values of different ointment bases formulations at changed temperatures throughout 45 days.

Day	Temperature	Free fatty acid values (g/mol)			
		F5	F6	F8	F9
0^th^	34°C	1.36	1.38	1.32	1.16
	40°C	1.23	1.23	1.23	1.13
5^th^	34°C	1.33	1.32	1.36	1.23
	40°C	1.35	1.35	1.35	1.35
15^th^	34°C	1.49	1.46	1.48	1.59
	40°C	1.46	1.34	1.36	1.45
30^th^	34°C	1.38	1.18	1.18	1.38
	40°C	1.26	1.16	1.19	1.26
45^th^	34°C	1.48	1.48	1.48	1.48
	40°C	1.49	1.46	1.41	1.49

## Discussion

The present study aims to verify the hypothesis that cow ghee provides the better formation of ointment bases with different traditional bases. Cow ghee used in conventional medicine is reported to have a beneficial effect on wound healing. Thus, we selected Cow as an ointment base for the ointment preparation with different traditional bases [7].

Although cow ghee is a standard Ayurvedic remedy, its potential for use has yet to be thoroughly investigated due to its lower stability. Consequently, this research was carried out to examine the use of cow ghee as an ointment base in the topical dosage form. It might be utilized as a replacement for the current topical ointment base in the forthcoming. Combining cow ghee with herbal medications or phytoconstituents as a base with traditional ointment bases might produce an effective herbal formulation. In this research, we prepared ointment bases with cow ghee having different concentrations from conventional bases, and their stability was also measured. All the required ointment bases are procured, and a routine Pharmacopoeia procedure was used to recognize and authenticate it. Cetostearyl alcohol, stearic acid, glyceryl monostearate, soft white paraffin, soft yellow paraffin, paraffin wax, white beeswax, and wool fat are all ointment bases selected. Based on certain factors, a routine or Pharmacopoeia procedure was used to recognize and authenticate cow ghee [2].

The parameters include solubility, pH, loss on drying, ash content, refractive index, specific gravity, saponification value, acid value, iodine value, moisture content, and peroxide value, which was performed for cow ghee [8]. After that total of nine ointment base formulation was prepared [9]. F1 includes Cetostearyl alcohol and cow ghee, having a 1:1 concentration. F2 includes stearic acid and cow ghee, having a 1:1 concentration. F3 includes glyceryl monostearate and cow ghee having a 1:1 concentration. F4 includes soft white paraffin and cow ghee having a 1:1 concentration. F5 includes soft yellow paraffin and cow ghee having 1:1 concentration. F6 includes soft yellow paraffin and cow ghee having 1:2 concentration. F7 includes soft yellow paraffin and cow ghee having 1:3 concentration. F8 includes soft white paraffin and cow ghee having a 1:2 concentration. F9 includes soft white paraffin and cow ghee with a 1:3 concentration [9].

Prepared all formulations of ointment bases are tested for their physical and chemical stability by studying various parameters like color, odor, consistency, pH, spreadability, washability, non-irritancy, and stability study at multiple temperatures (2°C, 25°C, and 37°C) [6]. This also performed chemical stability of prepared ointment bases, i.e., tested the free fatty acid level of all prepared formulated ointment bases. The study showed that ready ointment bases are physically and chemically stable. The findings from ointment base formulations F5, F6, F8, and F9 were superior in texture and spreadability to the other combinations of formulated ointment bases. Also, these ointment bases formulation was chemically stable, and there were no significant changes in free fatty acid level value.

## Conclusions

It is possible to prepare ointment bases containing cow ghee as a base with conventional bases; the prepared ointment bases were physically and chemically stable for at least 45 days at various temperatures like 2^o^C, 25^o^C, and 37^o^C. The findings from ointment base formulations F5, F6, F8, and F9 were superior in texture and spreadability to the other combination of formulated ointment bases. Also, these ointment bases formulation was chemically stable, and there were no significant changes in free fatty acid level value. 
